# COVID-19 and Ophthalmology: A New Chapter in an Old Story

**Published:** 2020-03-04

**Authors:** Victor Eduardo Reviglio, Matias Osaba, Virginia Reviglio, Pablo Chiaradia, Irene C. Kuo, Terrence P. O’Brien

**Affiliations:** 1 Instituto de la Vision Cerro de las Rosas, Sanatorio Allende – Sede Cerro, Cordoba, Argentina; 2 Facultad de Ciencias de la Salud, Universidad Catolica de Cordoba, Cordoba, Argentina; 3 Department of Ophthalmology, Hospital de Clinicas, School of Medicine, Universidad de Buenos Aires (UBA), Buenos Aires, Argentina; 4 Wilmer Eye Institute, Johns Hopkins University School of Medicine, Baltimore, USA; 5 Bascom Palmer Eye Institute, University of Miami Miller School of Medicine, Palm Beach Gardens, FL, USA

**Keywords:** Coronavirus, 2019-nCoV, Conjunctivitis, Ophthalmology, COVID-19

## INTRODUCTION

Recent reports disclose the fulminant illness and death of a young ophthalmologist named “Li Wenliang” at Wuhan Central Hospital in China following exposure with a glaucoma patient infected with coronavirus. The reportedly healthy ophthalmologist contracted coronavirus disease, COVID-19 (2019-nCoV) and succumbed a month later following an initially simple red eye associated to glaucoma [1].

This correspondence highlights a recent alert from the American Academy of Ophthalmology (AAO) regarding the new potentially pandemic caused by coronavirus 2019-nCoV and the risks associated to ophthalmic practice. Because increasing reports show that conjunctivitis may be the first presentation of coronavirus infection, triage by ophthalmological services may be the first line of transmission of 2019-nCoV as it can spread by aerosol contact with conjunctiva [2]. For this reason, the AAO and other officials “recommend protection for the mouth, nose and eyes when caring patients potentially infected with 2019-nCoV”. These recommendations include use of an N-95 mask and goggles or shield [3].

Since its emergence in December 2019, infection caused by 2019-nCoV has been characterized as a lower respiratory syndrome manifesting as pneumonia and/or acute respiratory distress. However, there are still considerable gaps in our knowledge of global epidemiology of 2019-nCoV, particularly as this infection is not confined to a geographical area and does not appear to be associated with socioeconomic status; however, its seasonal association remains unknown [4]. Previous influenza pandemics and outbreaks of other deadly infections including Ebola virus, Zika virus, chikungunya virus, dengue virus and malaria, have elicited an effective response from public health agencies, medical doctors and other healthcare professionals and both government and non-government agencies to control the spread of infection [2]. Today, we are facing a new virus demonstrating the ability to spread readily from close contact and across continents.

As ophthalmic physicians and surgeons, we have the scientific, medical and epidemiological knowledge of infectious agents causing ocular disease as well as systemic infection; adenovirus, and H1N1 are two other examples [5, 6]. Patients presenting to ophthalmology clinics or to the emergency room with conjunctivitis and possessing associated risk factor(s) (travel to high-risk areas or contact with persons who have returned from such areas or those who are known to be infected) can transmit 2019-nCoV infection even before they experience other signs and symptoms of infection. In other words, we ophthalmologists may be the first line of exposure as a newly-infected patient might seek care for acute conjunctivitis. 

Like Dr. Li Wenliang, we may be the first to diagnose a more serious condition as he did when he notified government officials and the public through social media after he visited seven patients of possible viral infection that presented similar to Severe Acute Respiratory Syndrome (SARS) [1]. Therefore, we ophthalmologists have an essential role in guiding infection control measures to prevent unnecessary exposures to other patients, staff and doctors [7]. Recommendations from the World Health Organization (WHO) [8], the Centers for Disease Control and Prevention (CDC) [2] in the United States, and public health agencies from other countries regarding standard precaution and safety measures for patients and healthcare professionals and protocols for limiting risk of spread are an important start against this fast-spreading potential pandemic. From previous experience with H1N1, pandemics can exert a highly disruptive impact on the operations of hospitals and outpatient clinics [5]. 

Anti-angiotensin-converting enzyme 2 (ACE2) antibody have been previously suggested to be able to block the coronavirus that causes severe acute respiratory syndrome (SARS-COV) replication on Vero E6 cells [9, 10]. It has been shown that ACE2 is a crucial SARS-CoV receptor in vivo, whereby injection of SARS-CoV Spike into mice worsened acute lung failure in vivo, which was attenuated by blocking the renin-angiotensin pathway [9, 10]. 

Also, it has been proposed that angiotensin II receptor blockers (ARB) is effective in the treatment of viral spread of novel coronavirus (Wuhan) [11], but more detailed clinical trials are needed to confirm the efficacy of different types of ARB on 2019-nCoV to prevent its outbreak.

As ophthalmologists, are we and our medical centers prepared to detect acute conjunctivitis due to 2019-nCoV? Do we have the necessary infrastructure for rapid triage, diagnosis, isolation and treatment of patients with 2019-nCoV? Do we have the necessary personal protection equipment to protect ourselves and to prevent the spread of infection? In potential pandemic emergencies like this one, we ophthalmologists must be vigilant, avail ourselves and our staff of task-specific education and training regarding prevention of spread, and comply with guidelines established by agencies such as WHO [8], and CDC [2]. A new chapter is being written today in the history of global infectious diseases. It is up to us ophthalmologists to assume our role and decide how we want to write the next ones.

## DISCLOSURE

Ethical issues have been completely observed by the authors. All named authors meet the International Committee of Medical Journal Editors (ICMJE) criteria for authorship of this manuscript, take responsibility for the integrity of the work as a whole, and have given final approval for the version to be published. No conflict of interest has been presented. Funding/Support: None. 
